# Surge of immune cell formation at birth differs by mode of delivery and infant characteristics—A population-based cohort study

**DOI:** 10.1371/journal.pone.0184748

**Published:** 2017-09-14

**Authors:** Titus Schlinzig, Stefan Johansson, Olof Stephansson, Lennart Hammarström, Rolf H. Zetterström, Ulrika von Döbeln, Sven Cnattingius, Mikael Norman

**Affiliations:** 1 Division of Pediatrics, Department of Clinical Science, Intervention and Technology, Karolinska Institutet, Stockholm, Sweden; 2 Department of Pediatric Perioperative Medicine and Intensive Care, Karolinska University Hospital, Stockholm, Sweden; 3 Department of Clinical Science and Education, Södersjukhuset (Karolinska Institutet SÖS), Stockholm, Sweden; 4 Department of Medicine Solna, Clinical Epidemiology Unit, Karolinska Institutet, Stockholm, Sweden; 5 Division of Obstetrics and Gynecology, Department of Women’s and Children’s Health, Karolinska Institutet, Stockholm, Sweden; 6 Division of Clinical Immunology, Department of Laboratory Medicine, Karolinska Institutet, Stockholm, Sweden; 7 Centre for Inherited Metabolic Diseases, Karolinska University Hospital, Stockholm, Sweden; 8 Department of Molecular Medicine and Surgery, Karolinska Institutet, Stockholm, Sweden; 9 Department of Medical Biochemistry and Biophysics, Division of Molecular Metabolism, Karolinska Institutet, Stockholm, Sweden; 10 Department of Neonatal Medicine, Karolinska University Hospital, Stockholm, Sweden; Centre Hospitalier Universitaire Vaudois, FRANCE

## Abstract

**Background:**

Birth by cesarean section is associated with increased risks of immune disorders. We tested whether establishment of immune function at birth relates to mode of delivery, taking other maternal and infant characteristics into account.

**Methods and findings:**

Using a prospectively collected database, we retrieved information on maternal and infant characteristics of 6,014 singleton infants delivered from February to April 2014 in Stockholm, Sweden, with gestational age ≥35 weeks, Apgar scores ≥7, and without congenital malformations or any neonatal morbidity. We linked our data to blood levels of T-cell receptor excision circles (TREC) and κ-deleting recombination excision circles (KREC), determined as part of a neonatal screening program for immune-deficiencies, and representing quantities of newly formed T- and B-lymphocytes. Multivariate logistic regression was used to calculate odds ratios (OR) with 95% confidence intervals (CI) for participants having TREC and KREC levels in the lowest quintile. Multivariate models were adjusted for postnatal age at blood sampling, and included perinatal (mode of delivery, infant sex, gestational age, and birth weight for gestational age), and maternal characteristics (age, parity, BMI, smoking, diabetes, and hypertensive disease).

Low TREC was associated with cesarean section before labor (adjusted OR:1.32 [95% CI 1.08–1.62]), male infant sex (aOR:1.60 [1.41–1.83]), preterm birth at 35–36 weeks of gestation (aOR:1.89 [1.21–2.96]) and small for gestational age (aOR:1.67 [1.00–2.79]). Low KREC was associated with male sex (aOR:1.32 [1.15–1.50]), postterm birth at ≥42 weeks (aOR:1.43 [1.13–1.82]) and small for gestational age (aOR:2.89 [1.78–4.69]). Maternal characteristics showed no consistent associations with neonatal levels of either TREC or KREC.

**Conclusion:**

Cesarean section before labor was associated with lower T-lymphocyte formation, irrespective of maternal characteristics, pregnancy, and neonatal risk factors. The significance of a reduced birth-related surge in lymphocyte formation for future immune function and health remains to be investigated.

## Introduction

The worldwide rate of Cesarean section (CS) has quadrupled in less than two decades, making CS the most common surgical procedure performed in women of child-bearing age [1, 2]. Whereas CS is judged as medically indicated in up to 20% of deliveries [3, 4], several national CS-rates exceed this level, suggesting that many women probably undergo CS without a clear medical indication [5]. Whereas short-term maternal and infant outcomes after CS are well described [6], the long-term consequences of this global change in mode of childbirth are mostly unknown.

Compared with vaginal delivery, CS delivery is associated with increased risks of immune disorders later in life, such as asthma and allergies [7–10], type 1 diabetes [10, 11], celiac disease and inflammatory bowel diseases [10, 12–14], obesity [15], immune deficiencies, leukemia, and other malignancies affecting young people [10, 16–18]. It is, however, unclear if and how CS could affect the health in the offspring [19, 20]. Possible pathways for these associations may include lower and untimely activation of the fetal immune system due to absence of labor with reduced stress of being born, and altered bacterial colonization of the infant gut after CS [21]. As elective cesarean delivery is almost exclusively performed before term gestation, lower gestational age has also been suggested to be in the causal pathway between CS and childhood and adult immune disease [19].

We have previously discovered that hematopoietic stem cells (CD34+) could have a cellular memory of birth, expressed as an epigenetic imprint [22]. Accordingly, DNA from stem cells in infants delivered by CS before labor was more methylated than DNA from infants delivered vaginally, indicating less genomic stem cell activation after CS than after vaginal delivery [22]. Whether epigenetic expressions translate into differential cellular actions remains to be established, however, we found differential DNA-methylation related to mode of delivery in regions of genes involved in immunoglobulin synthesis [22]. It is known that hematopoietic stem cells are important for the functional maturation of the immune system. Through differentiation, they give rise to mature T- and B-lymphocytes. At birth, the rate of stem cell differentiation peaks, after which a gradual decline occurs. Individual variations are substantial, and in the neonatal period, the quantity of newly formed lymphocytes can vary up to a 100-fold [23]. The underlying contributors to this variation in neonatal immune cell activation and differentiation are unknown.

Quantitative data on T-cell receptor excision circles (TRECs) and κ-deleting recombination excision circles (KRECs) are used as part of a neonatal screening program for detection of primary immune deficiencies [24] and reflect de novo formation of T- and B-lymphocytes from hematopoietic stem cells. In a large population-based cohort, we used this information to disentangle contributions from mode of delivery, infant and maternal characteristics to neonatal lymphocyte production after birth. Given how the pattern of DNA-methylation in lymphocyte ancestors relates to mode of delivery, our primary hypothesis was that newborn infants delivered by CS before labor exhibit lower levels of newly formed T- and B-lymphocytes (decreased levels of TRECs and KRECs) than infants born vaginally, irrespective of other perinatal factors. We also hypothesized that T- and B-cell formation could be affected by fetal growth and gestational age.

## Methods

### Study population

**Fig 1:**
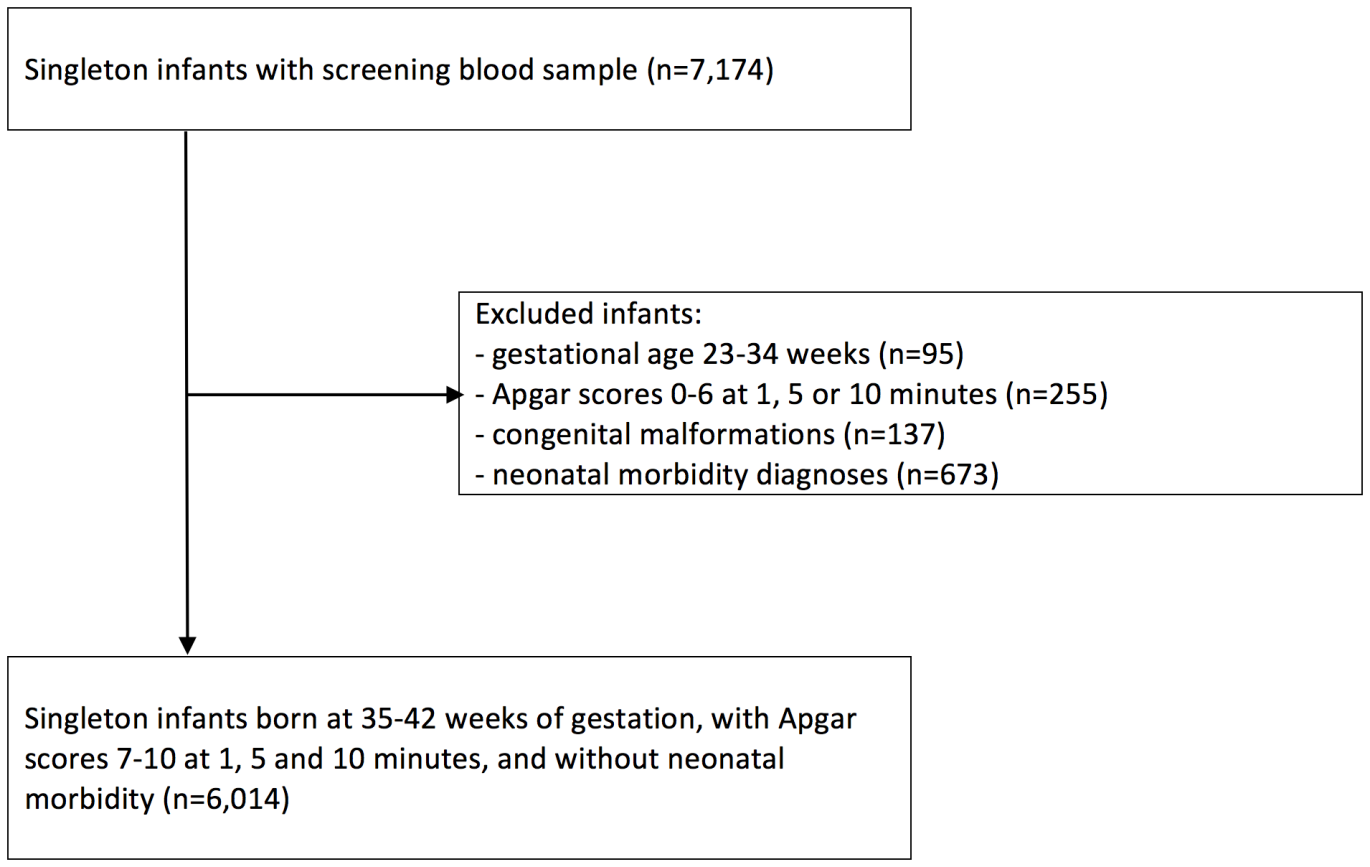
Study population of 6,014 live-born singleton infants born at 35–42 completed weeks of gestation.

This population-based cohort was based on 7,174 singleton, live-born infants, delivered in February through April 2014 in Stockholm county, Sweden. We restricted the study population to infants born at 35 completed weeks of gestation and later, with Apgar scores ≥7 at 1, 5 and 10 minutes, and without any record of ICD-10 diagnoses for congenital malformations or neonatal morbidity (the Q- and P-chapters in ICD-10). After these exclusions of 1,160 infants, the study population comprised 6,014 infants and their mothers, Fig 1.

Using the Stockholm-Gotland Obstetric Data Base, we retrieved maternal, pregnancy, delivery and infant characteristics. Information in this data base was prospectively collected; from the first antenatal visit that occurred during the first 13 gestational weeks in 90% of pregnancies, until postpartum hospital discharge.

### Main exposure

Mode of delivery was categorized as non-instrumental vaginal delivery, instrumental vaginal delivery, emergency or elective CS. Elective CS was defined as CS before onset of labor.

#### Other perinatal covariates

Maternal age was computed from the national identification number (mother’s birth date) at the date of delivery. Parity and self-reported smoking habits were based on information obtained at the first antenatal visit. Maternal body mass index (BMI; kg/m^2^) in early pregnancy was calculated from self-reported height and measured weight at the first antenatal visit. Maternal diabetes or hypertensive disease was defined as recorded ICD-10 diagnoses: diabetes included pre-gestational (ICD-10 codes E10-E14 and O240-O243) and gestational diabetes (ICD-10 code O244). Hypertensive diseases included pre-gestational (ICD-10 codes I10-I15, O10, O11) and gestational hypertension (ICD-10 code O13), and preeclampsia (ICD-10 codes O14-O15). Gestational age was determined by the following hierarchy: a) date of embryo transfer (2.8%), b) early second trimester ultrasound (96.1%), c) date of last menstrual period (1.1%), and d) from a postnatal assessment (<0.1%). Birth weight for gestational age was estimated according to the sex-specific Swedish reference curve for normal fetal growth. A normal birth weight for gestational age was defined as a birth weight between the 3rd and 97th percentiles. Postnatal age at blood sample was calculated by subtracting sampling date and time from birth date and time.

Perinatal covariates were categorized as presented in Table 1 and S1 Table.

**Table 1:** Perinatal characteristics of 6,014 singleton infants born at 35–42 weeks of gestation, and numbers and rates of TREC- and KREC-levels in the lowest quintile.

		Low TREC		p-value*	Low KREC		p-value*
	Total nb.	nb	rate (%)		nb	rate (%)	
**Mode of delivery**							
Elective C-section	640	176	(27.5)	*<0*.*01*	156	(24.4)	*0*.*02*
Emergency C-section	452	100	(22.1)		96	(21.2)	
Instrumental vaginal	325	58	(17.9)		64	(19.7)	
Non-instrumental vaginal	4597	869	(18.9)		887	(19.3)	
**Infant sex**							
Male	3068	728	(23.7)	*<0*.*01*	680	(22.2)	*<0*.*01*
Female	2946	475	(16.1)		523	(17.8)	
**Gestational age**							
35–36	96	34	(35.2)	*<0*.*01*	23	(24.0)	*<0*.*01*
37–41	5457	1078	(19.8)		1061	(19.4)	
42	461	91	(19.7)		119	(25.8)	
**Birth weight for gestational age**							
SGA, <3 perc	74	25	(33.8)	*0*.*03*	36	(48.7)	*<0*.*01*
AGA, 3–97 perc	5801	1153	(20.0)		1156	(20.0)	
LGA, >97 perc	137	24	(17.5)		11	(8.0)	
Missing	2	-	-		-	-	
**Postnatal age at blood sample**							
2	3147	809	(25.7)	*<0*.*01*	894	(28.4)	*<0*.*01*
3	1800	281	(15.6)		227	(12.6)	
4–10	1058	112	(10.6)		82	(7.8)	
Missing	9	1	-		1	-	

* According to chi square-test.

### Outcomes

The outcome variables, i.e. blood levels of TREC and KREC, were determined at the PKU laboratory at the Karolinska University Hospital, Stockholm, Sweden. TREC- and KREC-levels were analyzed as part of a neonatal screening project to detect severe combined immune deficiencies [24].

With minor technical modifications, the TREC/KREC newborn screening assay [23] was utilized. Using the original dried blood spots for the national neonatal screening program, TREC and KREC levels were determined in 3.2 mm punches. Every single 3.2-mm punch was analyzed in a 96-well format using quantitative triplex real-time qPCR for the simultaneous quantification of TRECs, KRECs and beta-actin (ACTB) using a ViiA7 Real-Time PCR System (Applied Biosystems, Foster City, CA, USA) as previously described [23]. Optimization of the qPCR procedure was performed using custom reagents provided by Affymetrix (Santa Clara, CA, USA). The purpose of the inclusion of ACTB in the assay was to assess that the extraction and amplification were satisfactory from each dried blood spot, but this was disregarded if TREC and KREC copy numbers were above the cutoff levels. Samples in which TREC or KREC levels were below cutoff in association with a reduction in ACTB copy number were considered inconclusive and were reanalyzed.

### Statistical analyses

Perinatal characteristics were described as numbers and rates, and compared with chi-squared test. Levels of TREC and KREC were log-transformed to achieve a normal distribution, and divided into quintiles, from low to high values. Chi-square test was used to estimate differences in distributions of exposure (mode of delivery and other perinatal covariates) in relation to TREC- and KREC-quintiles. In addition, linear regression models were used to investigate whether the log-transformed TREC- or KREC-values were associated with our perinatal covariates. We also used logistic regression to calculate odds ratios with 95% confidence intervals for the risk of having a TREC- or KREC-value within the lowest quintile (Q1), with the other quintiles (Q2-Q5) as the reference category. As postnatal age for blood sampling was strongly associated with TREC- and KREC-values, we investigated whether postnatal age for sampling modified associations. A statistical interaction was pre-defined as an interaction term with p-value of less than 0.05. Finally, sensitivity analyses were performed in the subset of infants with mothers without recorded diabetes and hypertensive disease.

The SAS software package version 9.4 (SAS Institute Inc., Cary, NC, USA) was used for statistical analyses. The study was approved by the Regional Ethical Review Board in Stockholm, Sweden (No. 2014/1292-31/4). Informed consent to the study was waived as all participants previously had agreed on participation in the neonatal screening program and to data collection in the Stockholm-Gotland database, both for clinical and research purposes.

## Results

Neonatal blood levels of TREC ranged from 16–622 copies/3,2 mm punch with a median value of 166 copies/3,2 mm punch; KREC blood levels ranged from 2–638 copies/3,2 mm punch, with a median of 97 copies/3,2 mm punch.

**Fig 2:**
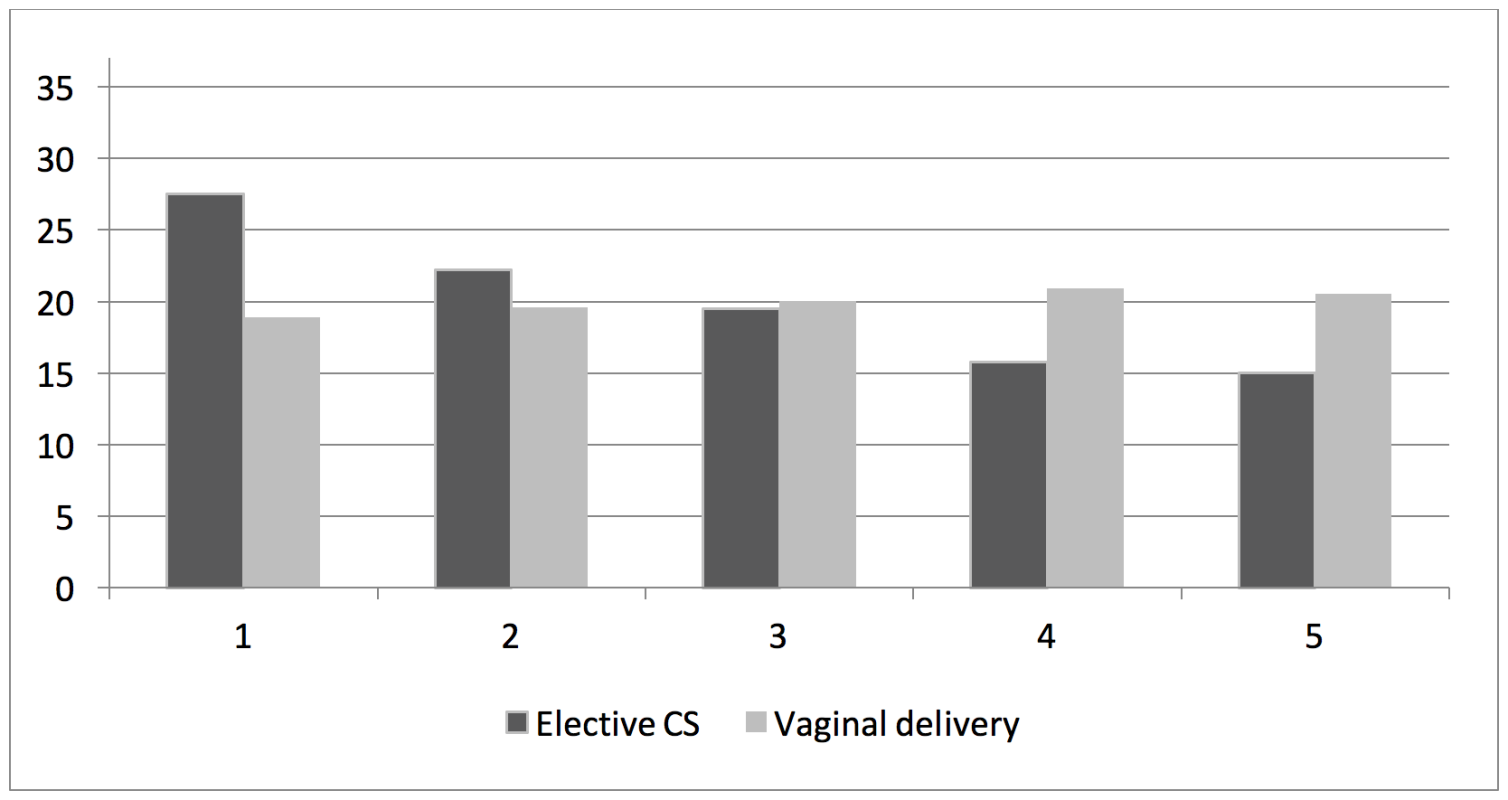
Rates (%) of TREC values in quintiles (1 = lowest quintile) after elective caesarean section and non-instrumental vaginal delivery, respectively. (Chi-square p-value<0.001).

**Fig 3:**
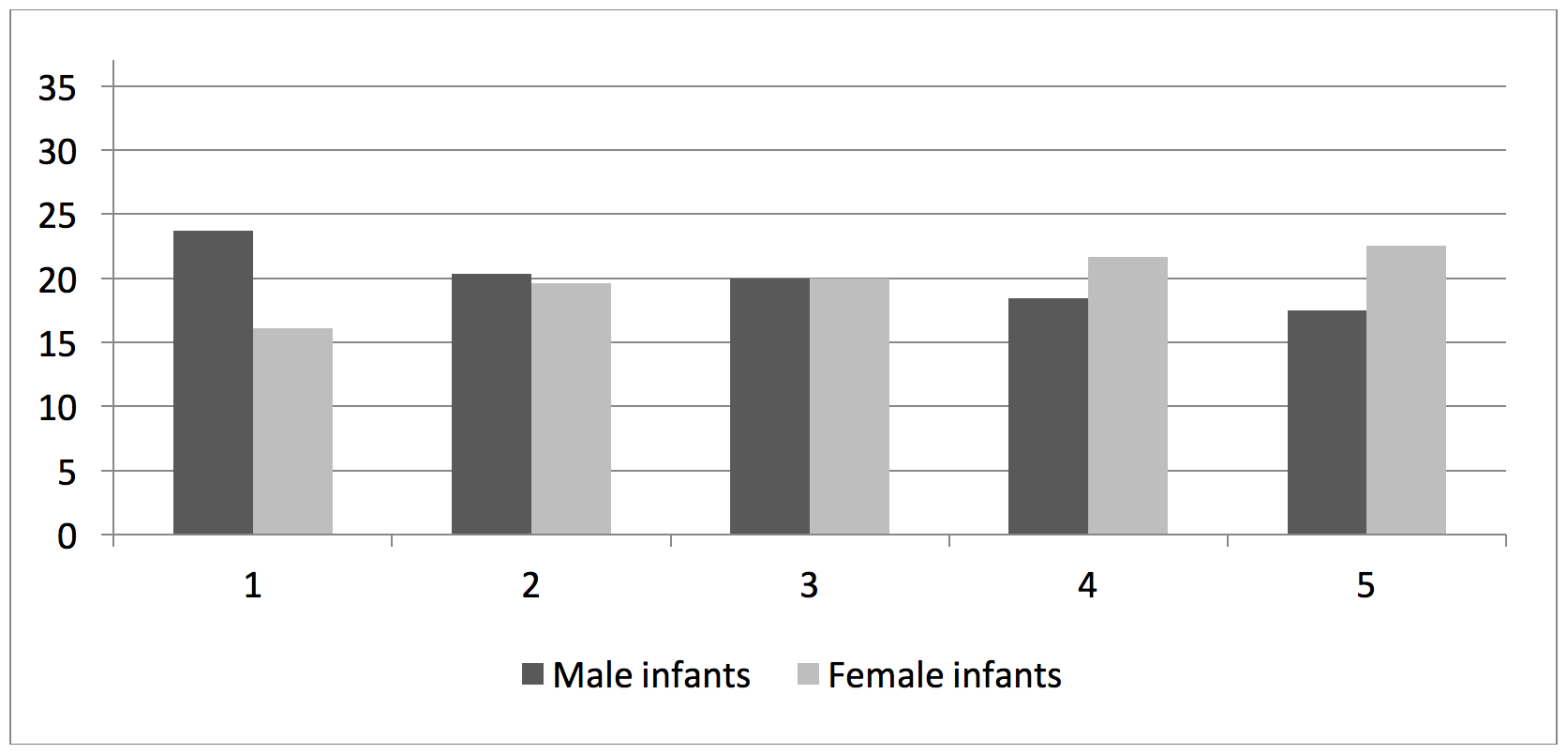
Rates (%) of TREC values in quintiles (1 = lowest quintile) in male and female infants, respectively. (Chi-square p-value<0.001).

**Fig 4:**
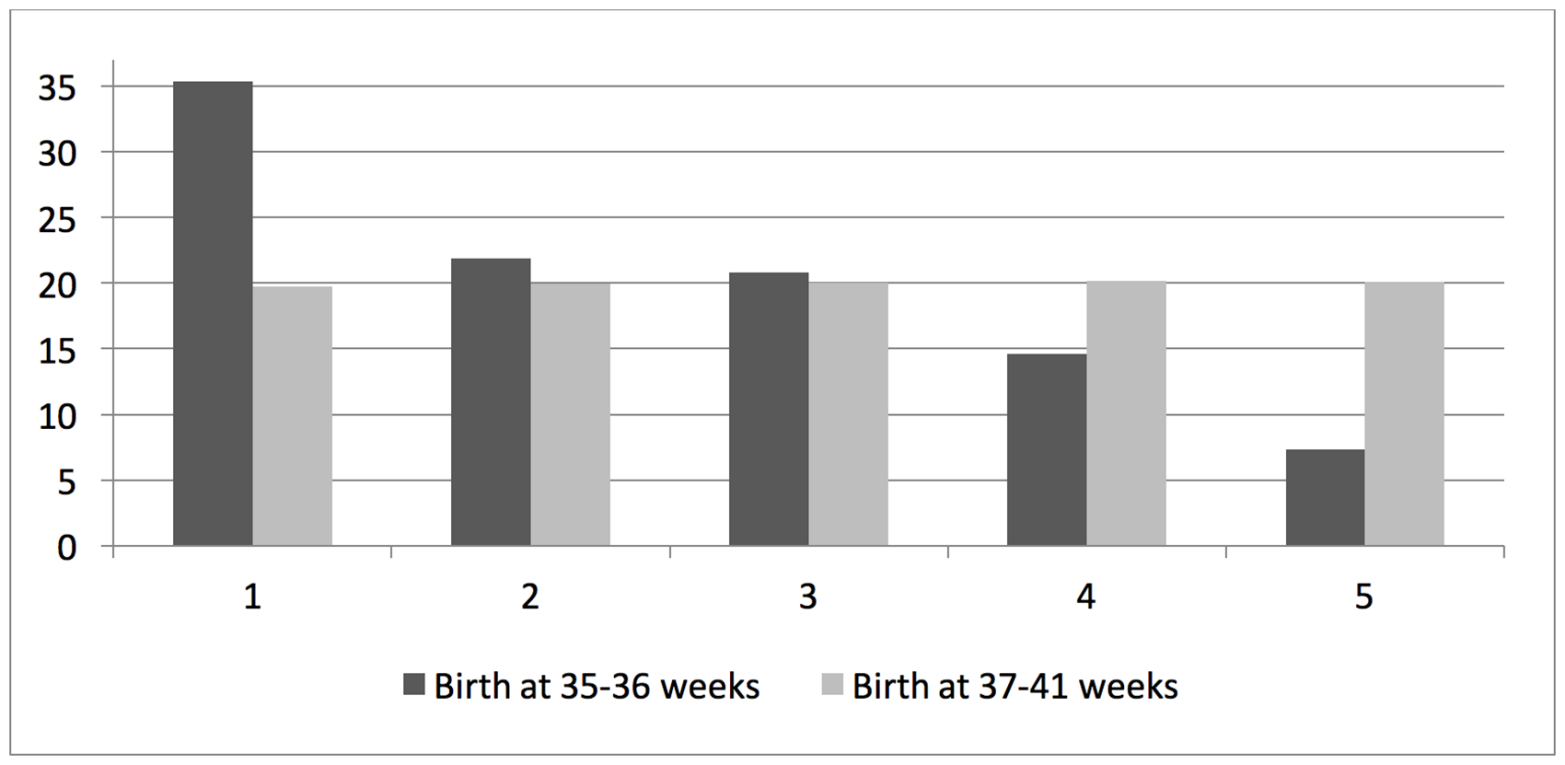
Rates (%) of TREC values in quintiles (1 = lowest quintile) after preterm birth (35–36 weeks) and term birth (37–41 weeks), respectively. (Chi-square p-value<0.001).

**Fig 5:**
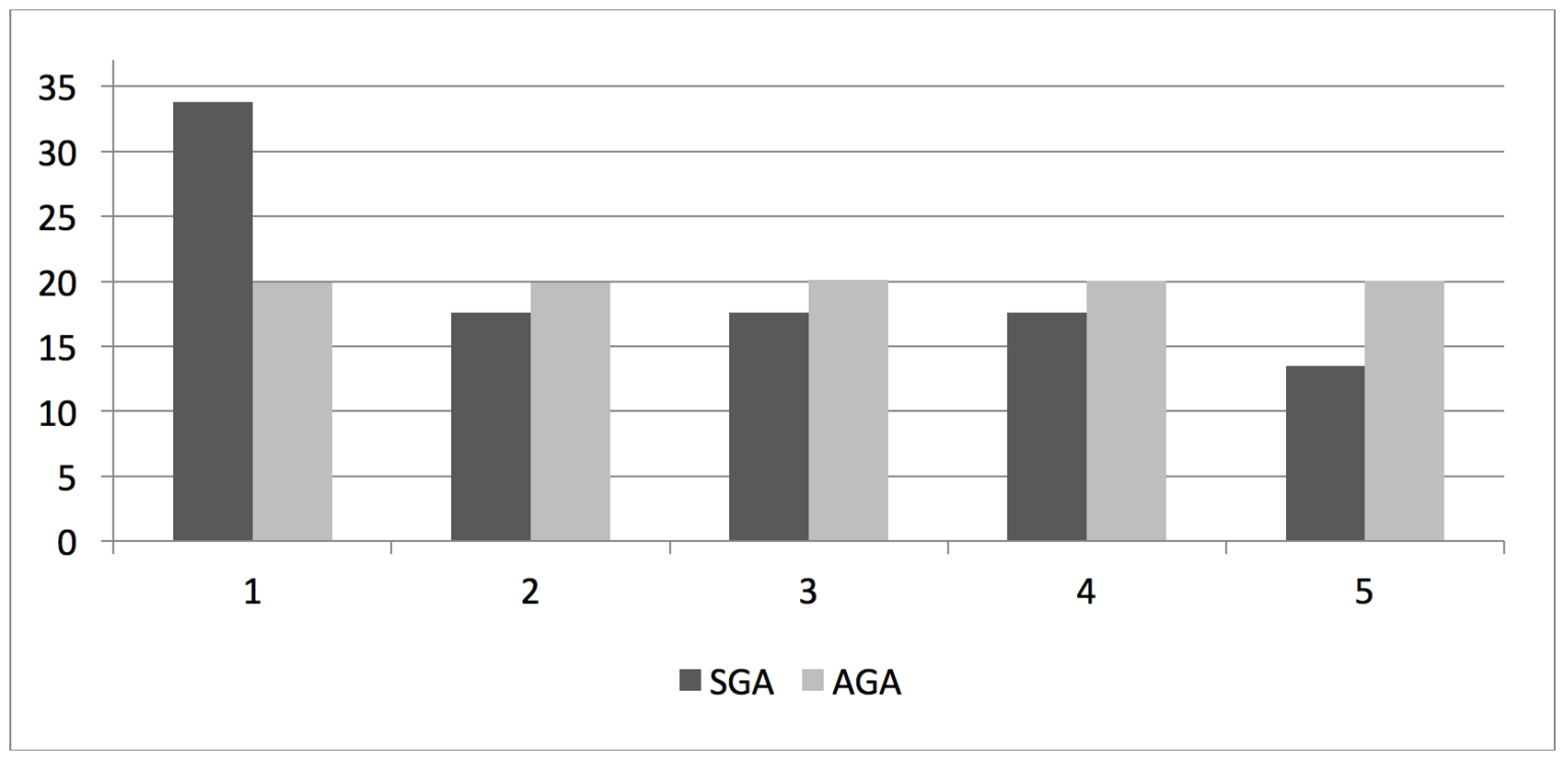
Rates (%) of TREC values in quintiles (1 = lowest quintile) in infants with low (SGA) and appropriate (AGA) birth weights for gestational age, respectively. (Chi-square p-value = 0.05).

In the study population of 6,014 singleton infants born between 35–42 gestational weeks, low TREC- and KREC-levels (within the first quintile) were more commonly found after elective CS, in male infants, after preterm birth (35–36 weeks), in infants with low birth weight for gestational age, and when the blood sample was taken within the first 3–4 postnatal days (Table 1). In addition, the distributions of TREC-levels were left-shifted (towards lower levels) in infants delivered by elective CS compared with those delivered vaginally, in male compared to female infants, after preterm (35–36 weeks) compared to term birth (37–41 weeks), and in infants with low birth weight for gestational age as compared to infants with appropriate birth weight for gestational age (Figs 2–5). In contrast, rates of low TREC and KREC did not differ by maternal characteristics, such as age, parity, BMI and smoking (S1 Table). However, the rate of a low TREC-level was higher in infants to diabetic mothers, and the rate of a low KREC-level was higher in infants to mothers with hypertensive disease.

The proportions of infants with TREC- and KREC-levels in the lowest quintile decreased with infant postnatal age and therefore, all logistic regression models were adjusted for postnatal age at blood sampling.

In the adjusted models, elective CS, male infant sex, preterm birth at 35 to 36 weeks gestation and smallness for gestational age (SGA) were associated with increased odds for a TREC-level in the lowest quintile (Table 2). Male infants, infants born post-term (≥ 42 weeks of gestation) and SGA-infants had increased odds for low KREC-levels, whereas elective CS was not associated with increased risk for low KREC (Table 2). Maternal characteristics showed no consistent associations with risks of low TREC or KREC levels (S2 Table). As mode of delivery may be related to gestational age, we tested whether gestational age modified associations between mode of delivery and low TREC and low KREC, respectively. However, gestational age did not interact with mode of delivery (interaction terms p = 0.29 and p = 0.97, respectively).

**Table 2:** Risks of a low TREC- and KREC-levels related to perinatal characteristics of 6,014 singleton infants born at 35–42 weeks of gestation.

	Low TREC		Low KREC	
	Odds Ratio (95% confidence interval)		Odds Ratio (95% confidence interval)	
	Crude	Adjusted*	Crude	Adjusted*
**Mode of delivery**				
Elective C-section	1.63 (1.35–1.97)	1.32 (1.08–1.62)	1.35 (1.11–1.64)	1.13 (0.91–1.39)
Emergency C-section	1.22 (0.96–1.54)	0.95 (0.74–1,22)	1.13 (0.89–1.43)	0.75 (0.58–0.97)
Instrumental vaginal	0.93 (0.70–1.25)	0.87 (0.64–1.17)	1.03 (0.77–1.36)	0.80 (0.59–1.08)
Non-instrumental vaginal	1.00 (ref.)	1.00 (ref.)	1.00 (ref.)	1.00 (ref.)
**Infant sex**				
Male	1.62 (1.42–1.84)	1.60 (1.41–1.83)	1.32 (1.16–1.50)	1.32 (1.15–1.50)
Female	1.00 (ref.)	1.00 (ref.)	1.00 (ref.)	1.00 (ref.)
**Gestational age**				
35–36	2.23 (1.46–3.40)	1.89 (1.21–2.96)	1,31 (0.81–2.10)	1.07 (0.65–1.75)
37–41	1.00 (ref.)	1.00 (ref.)	1.00 (ref.)	1.00 (ref.)
42	1.00 (0,79–1.27)	0.99 (0.77–1.28)	1.44 (1.16–1.80)	1.43 (1.13–1.82)
**Birth weight for gestational age**				
SGA, <3 perc	2.06 (1.27–3.34)	1.67 (1.00–2.79)	3.81 (2.4–6.03)	2.89 (1.78–4.69)
AGA, 3–97 perc	1.00 (ref.)	1.00 (ref.)	1.00 (ref.)	1.00 (ref.)
LGA, >97 perc	0.86 (0.55–1.34)	0.81 (0.51–1.28)	0.35 (0.19–0.65)	0.37 (0.20–0.70)
**Postnatal day at blood sample**				
2	1.00 (ref.)	1.00 (ref.)	1.00 (ref.)	1.00 (ref.)
3	0.54 (0.46–0.62)	0.55 (0.47–0.64)	0.36 (0.31–0.43)	0.38 (0.32–0.45)
4–10	0.34 (0.28–0.42)	0.34 (0.27–0.42)	0.21 (0.17–0.27)	0.22 (0.17–0.28)

* Adjusted for perinatal characteristics (mode of delivery, infant sex, gestational age, birth weight for gestational age and postnatal age at blood sample) and for maternal characteristics (age, parity, BMI, smoking, diabetes, and hypertensive disease). Crude and adjusted odds ratios for maternal characteristics are presented in S2 Table.

Given the strong and inverse association between postnatal age and low TREC- and KREC-levels, we investigated whether postnatal day for blood sampling modified the associations between other perinatal covariates with regard to risks of low TREC- or KREC-levels. However, we found no statistical interactions between postnatal day for blood sampling and mode of delivery, gestational age, infant sex or birth weight for gestational age, respectively.

Since rates of low TREC- and KREC-values were higher in infants to mothers with diabetes and hypertensive disease (S1 Table), we repeated the logistic regression analyses within the subset of 5,681 infants to mothers without diabetes and hypertensive disease. Compared to the associations within the complete cohort, similar results were found in these sub-analyses (S3 Table).

## Discussion

In this study, we found that de novo production of neonatal lymphocytes was associated with mode of delivery and other perinatal characteristics. Compared to vaginal delivery, CS was associated with 32% higher risk of having an infant with a lower number of newly formed T-lymphocytes (TREC values within the lowest quintile). In addition, male sex, delivery at 35 to 36 gestational weeks, and being born SGA were also associated with a shift towards lower T-lymphocyte formation. Infants born SGA were also at increased risk of low levels of B-lymphocytes (KREC values within the lowest quintile) compared to infants with normal birth weight for gestational age. Associations between KREC values and mode of delivery or other infant characteristics were less pronounced or absent. Importantly, maternal characteristics or complications during pregnancy were not associated with levels of TRECs or KRECs in the offspring. To our knowledge, these associations have not been previously reported and the selective contributions from each of these risk factors to the neonatal immune phenotype have not been possible to disentangle before.

Birth has previously been associated with acute leukocyte recruitment [25–29]. This establishment of immune function is thought to be mediated by the stress of being born. With progressive increase in fetal stress during delivery, significant cord blood elevations in total number of white blood cells as well as increased levels of IL-8, soluble E-Selectin and Interferon-c levels were reported [30, 31]. Accordingly, labor and birth stress may act as primers and regulators of the neonatal immune system. Given that stress of being born is strongly related to mode of delivery, with much lower sympatho-adrenal activation after elective CS than after vaginal delivery [32, 33], mode of delivery appears to be an important determinant of the postnatal adaption of the offspring immune system [30].

The present findings extend our knowledge on how mode of delivery affects the immune system. We found that elective CS was associated with a reduced differentiation into T-lymphocytes after birth. Whether or not this is just a transient neonatal phenomenon remains to be established. Differentiation of immature T-, and B-cells gives rise to a pool of long-lived lymphocytes [34]. If this pool is reduced already from start, it may have clinical implications at a later stage, particularly when the growing individual becomes exposed to infectious agents and antigens. Even if there could be other explanations besides a poor activation of the neonatal immune system such as a perturbation of the gut microbiome [21], we note that birth by CS increases the risk of gastroenteritis in childhood [35].

Accumulating evidence links early fetal-neonatal living conditions to later health in adult life [36]. Recent epidemiological studies have confirmed that CS is associated with a moderately increased risk for immune disorders later in life, also after taking potential confounders into account [10]. The mechanisms behind such early imprints are still largely unclear. As indicated herein, reduced differentiation into effector or memory T-cells after CS could be one option. Other data indicate an epigenetic origin. In a previous study of neonatal hematopoietic stem cells, i.e., the precursors of T- and B-cells, we found differential DNA-methylation in relation to mode of delivery [22]. Interestingly, locus-specific differences in DNA-methylation related to mode of delivery included at least one gene associated with a genetic predisposition for diabetes type 1 [22].

In addition to mode of delivery, we identified male sex as a risk factor for low TRECs after birth. Consistent with our findings, cord blood from female infants contains a higher number of CD4+ T-lymphocytes, higher CD4/CD8 T-lymphocyte ratios and lower numbers of CD8+ T-lymphocytes and NK cells than cord blood from male infants, whereas the number of B- lymphocytes is comparable in males and females [37, 38]. Sex differences related to immune disorders have also been shown previously [39]. Diabetes type 1 [40], asthma [41, 42], hypertensive disease [42] and inflammatory bowel disease [43] mostly affect males more than females. A male disadvantage in immune responses to infectious diseases [44] and outcome after sepsis [45] has also been demonstrated.

Preterm born infants have functional deficiencies in their immune system and are more vulnerable to infections [46]. The fundamental mechanisms of the innate immune system and how immaturity contributes to the overall risk of infection during the neonatal period are not completely understood, but preterm birth is considered the largest risk factor for infections during the neonatal period [47]. In this study we demonstrated that also near-term birth, i.e., at 35–36 weeks, was associated with a significant reduction in T-lymphocytes. We also found strong associations between smallness for gestational age and low TRECs and KRECs values, respectively. Previous studies reported that infants born small for gestational age have a significantly smaller number of T- and B-lymphocytes [48] and constitutional small for gestational age fetuses had a disproportionately small thymus [49]. The most obvious implication of these findings is that gestational age and fetal growth should be taken into account when studying associations between mode of delivery and structure and function of the neonatal immune system, as well as associations between mode of delivery and later health-related outcomes. Moreover, we note that low birth weight has been identified as a risk factor for adult diseases where inflammation plays a key role, namely hypertension, coronary heart disease, dyslipidemia, type 2 diabetes and obesity [50–53].

Strengths of this study include its population-based design, minimizing selection bias and ensuring generalizability to pregnant women and their infants in high-resource countries like Sweden. We studied a relatively large cohort with numbers that exceeded most previous studies of the neonatal immune system, and with detailed exposure and outcome data. The sample size enabled us to adjust for potential confounders, and to disentangle the selective contributions from important covariates. The method for TREC and KREC determinations has been validated [23].

This study also has limitations. We measured TREC and KREC levels in blood representing the number of newly formed lymphocytes, but not in tissues. Stress induces a mobilization of lymphocytes into the blood, followed by a trafficking of these cells out of the blood into different body compartments [54]. Therefore, we cannot exclude that redistribution of lymphocytes could affect our results. Our database does not contain data on time of cord clamping and even if we have national and local guidelines instructing the staff to perform late cord clamping in all but emergency cases (and irrespective of mode of delivery), we cannot exclude that timing of cord clamping—and thereby postnatal placental transfusion of blood cells to the infant—may have differed in relation to mode of delivery. Although analyses were adjusted for a range of potential confounders, and sensitivity analyses after excluding mothers with diabetes or hypertensive diseases did not alter our results, we cannot exclude the possibility of residual confounding. We acknowledge that levels of TREC and KREC changed over time from birth and therefore adjusted for postnatal age at blood sampling but we lacked longitudinal data beyond the first week of life to determine if our findings represent a transient phenomenon or a lasting difference in the lymphocyte pool. Our data did not permit any exploration of innate immunity, tolerance or microbiota colonization, and we missed information on any autoimmune disease or inflammatory status in the mother during pregnancy. Finally, short- or long-term clinical implications of our findings are unclear.

In conclusion, this study provides another piece of knowledge on establishment of immune function at birth and its relation to various perinatal risk factors, such as gestational age, mode of delivery and birth weight. The rapidly increasing rates of CS worldwide and the greater risk for immune disorders later in life add fuel to efforts trying to resolve these issues. Our study has identified several important risk factors for reduced number of T- and B-lymphocytes in newborn infants, and CS delivery without a clear medical indication may be the one that can be modified most rapidly. Future research on elective CS and health effects in offspring should focus on longitudinal aspects targeting lymphocyte numbers and function, as well as on how low neonatal lymphocyte numbers relate to infections as well as disorders related to immune function later in life.

## Supporting information

S1 Table Maternal characteristics of 6,014 singleton live-birth at 35–42 weeks of gestation, and numbers and rates of TREC- and KREC-levels in the lowest quintile. (DOCX)

S2 Table Risks of a low TREC- and KREC-levels related to maternal characteristics of 6,014 singleton live-births at 35–42 weeks of gestation. (DOCX)

S3 Table Risks of a low TREC- and KREC-levels related to characteristics of 5,681 singleton infants born at 35–42 weeks of gestation, to mothers without diabetes or hypertensive disease. (DOCX)

## Abbreviations

ACTB: Actin beta
AGA: appropriate for gestational age
BMI: body mass index
CS: cesarean section
DNA: Deoxyribonucleic Acid
ICD: international classification of disease
KRECs: κ-deleting recombination excision circles
LGA: large for gestational age
PKU: phenylketonuria
qPCR: quantitative polymerase chain reaction
SGA: small for gestational age
TRECs: T-cell receptor excision circles
