# Supplementary material for: Surge of immune cell formation at birth differs by mode of delivery and infant characteristics—A population-based cohort study
Source: PLoS One. 2017 Sep 14;12(9):e0184748. doi: 10.1371/journal.pone.0184748 (PMC5599043; doi:10.1371/journal.pone.0184748)
Supplement: S1 Table — (DOCX) [file pone.0184748.s001.docx]

**S1 Table. Maternal characteristics of 6,014 singleton live-birth at 35-42 weeks of gestation, and numbers and rates of TREC- and KREC-levels in the lowest quintile.**

|  |  | **Low TREC** | |  | **Low KREC** | |  |
| --- | --- | --- | --- | --- | --- | --- | --- |
|  | **Total nb.** | **nb** | **rate (%)** | *p-value** | **nb** | **rate (%)** | *p-value** |
| **Maternal age (years)** |  |  |  |  |  |  |  |
| <25 | 502 | 99 | (19.7) | *0.49* | 121 | (24.1) | *0.01* |
| 25-29 | 1584 | 327 | (20.6) |  | 292 | (18.4) |  |
| 30-34 | 2324 | 447 | (19.2) |  | 488 | (21.0) |  |
| 35-39 | 1311 | 260 | (19.8) |  | 244 | (18.6) |  |
| ≥40 | 292 | 70 | (24.0) |  | 57 | (19.5) |  |
| Missing | 1 | - | - |  | 1 | - |  |
|  |  |  |  |  |  |  |  |
| **Parity** |  |  |  |  |  |  |  |
| 1-para | 2512 | 511 | (20.3) | *0.84* | 591 | (23.5) | *<0.01* |
| 2-para | 2506 | 492 | (19.7) |  | 419 | (16.7) |  |
| ≥3-para | 996 | 199 | (20.0) |  | 193 | (19.4) |  |
|  |  |  |  |  |  |  |  |
| **BMI (kg/m^2^)** |  |  |  |  |  |  |  |
| <18.5 | 174 | 36 | (20.7) | *0.40* | 36 | (20.7) | *0.82* |
| 18.5-24.9 | 3953 | 766 | (19.4) |  | 791 | (20.0) |  |
| 25-29.9 | 1248 | 259 | (20.8) |  | 239 | (19.2) |  |
| ≥30 | 503 | 115 | (22.9) |  | 109 | (21.7) |  |
| Missing | 136 | 27 | - |  | 28 | - |  |
|  |  |  |  |  |  |  |  |
| **Smoking** |  |  |  |  |  |  |  |
| No | 5747 | 1148 | (20.0) | *0.80* | 1143 | (19.9) | *0.30* |
| Yes | 267 | 55 | (20.6) |  | 60 | (22.5) |  |
|  |  |  |  |  |  |  |  |
| **Diabetes** |  |  |  |  |  |  |  |
| No | 5965 | 1187 | (19.9) | *0.03* | 1194 | (20.0) | *0.77* |
| Yes | 49 | 16 | (32.7) |  | 9 | (18.4) |  |
|  |  |  |  |  |  |  |  |
| **Hypertensive Disease** |  |  |  |  |  |  |  |
| No | 5716 | 1137 | (20.0) | *0.34* | 1128 | (19.7) | *0.02* |
| Yes | 298 | 66 | (22.2) |  | 75 | (25.2) |  |

* According to chi square-test.
