# Supplementary material for: Surge of immune cell formation at birth differs by mode of delivery and infant characteristics—A population-based cohort study
Source: PLoS One. 2017 Sep 14;12(9):e0184748. doi: 10.1371/journal.pone.0184748 (PMC5599043; doi:10.1371/journal.pone.0184748)
Supplement: S2 Table — (DOCX) [file pone.0184748.s002.docx]

**S2 Table. Risks of a low TREC- and KREC-levels related to maternal characteristics of 6,014 singleton live-births at 35-42 weeks of gestation.**

|  | **Low TREC** | | **Low KREC** | |
| --- | --- | --- | --- | --- |
|  | **Odds Ratio (95% confidence interval)** | | **Odds Ratio (95% confidence interval)** | |
|  | **Crude** | **Adjusted*** | **Crude** | **Adjusted*** |
| **Maternal age (years)** |  |  |  |  |
| <25 | 0.94 (0.73-1.21) | 0.90 (0.69-1.17) | 1.41 (1.11-1.79) | 1.27 (0.98-1.64) |
| 25-29 | 1.00 (ref.) | 1.00 (ref.) | 1.00 (ref.) | 1.00 (ref.) |
| 30-34 | 0.92 (0.78-1.07) | 0.92 (0.78-1.09) | 1.18 (1.00-1.38) | 1.25 (1.05-1.48) |
| 35-39 | 0.95 (0.79-1.14) | 0.95 (0.78-1.16) | 1.01 (0.84-1.22) | 1.07 (0.87-1.32) |
| ≥40 | 1.21 (0.90-1.63) | 1.17 (0.85-1.60) | 1.07 (0.78-1.47) | 1.13 (0.80-1.58) |
|  |  |  |  |  |
| **Parity** |  |  |  |  |
| 1-para | 1.4 (0.91-1.20) | 0.90 (0.77-1.05) | 1.53 (1.33-1.76) | 1.17 (1.00-1.37) |
| 2-para | 1.00 (ref.) | 1.00 (ref.) | 1.00 (ref.) | 1.00 (ref.) |
| ≥3-para | 1.02 (0.85-1.23) | 0.95 (0.78-1.15) | 1.20 (0.99-1.47) | 1.19 (0.97-1.45) |
|  |  |  |  |  |
| **BMI (kg/m^2^)** |  |  |  |  |
| <18.5 | 1.09 (0.75-1.58) | 1.08 (0.73-1.59) | 1.04 (0.72-1.52) | 0.99 (0.67-1.46) |
| 18.5-24.9 | 1.00 (ref.) | 1.00 (ref.) | 1.00 (ref.) | 1.00 (ref.) |
| 25-29.9 | 1.09 (0.93-1.28) | 1.07 (0.91-1.26) | 0.95 (0.81-1.11) | 0.94 (0.79-1.11) |
| ≥30 | 1.23 (0.99-1.54) | 1.10 (0.87-1.40) | 1.11 (0.88-1.39) | 1.02 (0.80-1.29) |
|  |  |  |  |  |
| **Smoking** |  |  |  |  |
| No | 1.00 (ref.) | 1.00 (ref.) | 1.00 (ref.) | 1.00 (ref.) |
| Yes | 0.96 (0.71-1.30) | 1.06 (0.77-1.46) | 0.86 (0.64-1.15) | 0.94 (0.69-1.29) |
|  |  |  |  |  |
| **Diabetes** |  |  |  |  |
| No | 1.00 (ref.) | 1.00 (ref.) | 1.00 (ref.) | 1.00 (ref.) |
| Yes | 1.95 (1.07-3.56) | 1.56 (0.84-2.89) | 0.90 (0.44-1.86) | 0.74 (0.35-1.56) |
|  |  |  |  |  |
| **Hypertensive Disease** |  |  |  |  |
| No | 1.00 (ref.) | 1.00 (ref.) | 1.00 (ref.) | 1.00 (ref.) |
| Yes | 1.5 (0.87-1.52) | 0.92 (0.69-1.24) | 1.37 (1.05-1.79) | 1.12 (0.84-1.50) |

* Adjusted for perinatal characteristics (mode of delivery, infant sex, gestational age, birth weight for gestational age and postnatal age at blood sample) and for maternal characteristics (age, parity, BMI, smoking, diabetes, and hypertensive disease). Crude and adjusted odds ratios for perinatal characteristics are presented in Table 2.
