# Supplementary material for: Surge of immune cell formation at birth differs by mode of delivery and infant characteristics—A population-based cohort study
Source: PLoS One. 2017 Sep 14;12(9):e0184748. doi: 10.1371/journal.pone.0184748 (PMC5599043; doi:10.1371/journal.pone.0184748)
Supplement: S3 Table — (DOCX) [file pone.0184748.s003.docx]

**S3 Table. Risks of a low TREC- and KREC-levels related to characteristics of 5,681 singleton infants born at 35-42 weeks of gestation, to mothers without diabetes or hypertensive disease.**

|  | **Low TREC** | **Low KREC** |
| --- | --- | --- |
|  | **Adjusted* odds ratio**  **(95% confidence interval)** | **Adjusted* odds ratio**  **(95% confidence interval)** |
| **Mode of delivery** |  |  |
| Elective C-section | 1.36 (1.10-1.68) | 1.13 (0.91-1.41) |
| Emergency C-section | 0.95 (0.73-1.23) | 0.78 (0.60-1.02) |
| Instrumental vaginal | 0.88 (0.64-1.20) | 0.74 (0.54-1.02) |
| Non-instrumental vaginal | 1.00 (ref.) | 1.00 (ref.) |
|  |  |  |
| **Infant sex** |  |  |
| Male | 1.65 (1.44-1.89) | 1.33 (1.16-1.52) |
| Female | 1.00 (ref.) | 1.00 (ref.) |
|  |  |  |
| **Gestational age** |  |  |
| 35-36 | 2.00 (1.24-3.25) | 0.86 (0.48-1.51) |
| 37-41 | 1.00 (ref.) | 1.00 (ref.) |
| 42 | 0.95 (0.74-1.24) | 1.42 (1.11-1.81) |
|  |  |  |
| **Birth weight for gestational age** |  |  |
| SGA, <3 perc | 1.84 (1.07-3.18) | 2.99 (1.77-5.04) |
| AGA, 3-97 perc | 1.00 (ref.) | 1.00 (ref.) |
| LGA, >97 perc | 0.77 (0.47-1.28) | 0.39 (0.20-0.76) |
|  |  |  |
| **Postnatal age at**  **blood sample** |  |  |
| 2 | 1.00 (ref.) | 1.00 (ref.) |
| 3 | 0.54 (0.46-1.28) | 0.36 (0.30-0.43) |
| 4-10 | 0.32 (0.26-0.40) | 0.21 (0.16-0.27) |
|  |  |  |
| **Maternal age (years)** |  |  |
| <25 | 0.90 (0.69-1.19) | 1.27 (0.98-1.66) |
| 25-29 | 1.00 (ref.) | 1.00 (ref.) |
| 30-34 | 0.92 (0.77-1.09) | 1.27 (1.06-1.51) |
| 35-39 | 0.96 (0.78-1.18) | 1.08 (0.88-1.34) |
| ≥40 | 1.21 (0.87-1.67) | 1.19 (0.84-1.69) |
|  |  |  |
| **Parity** |  |  |
| 1-para | 0.94 (0.80-1.10) | 1.17 (1.00-1.38) |
| 2-para | 1.00 (ref.) | 1.00 (ref.) |
| ≥3-para | 0.94 (0.77-1.15) | 1.15 (0.94-1.42) |
|  |  |  |
|  |  |  |
|  |  |  |
|  |  |  |
| **BMI (kg/m^2^)** |  |  |
| <18.5 | 1.07 (0.70-1.35) | 0.97 (0.65-1.44) |
| 18.5-24.9 | 1.00 (ref.) | 1.00 (ref.) |
| 25-29.9 | 1.04 (0.88-1.23) | 0.92 (0.77-1.09) |
| ≥30 | 1.02 (0.79-1.31) | 0.92 (0.71-1.20) |
|  |  |  |
| **Smoking** |  |  |
| No | 1.00 (ref.) | 1.00 (ref.) |
| Yes | 0.97 (0.70-1.35) | 0.87 (0.63-1.19) |

* Adjusted for perinatal characteristics (mode of delivery, infant sex, gestational age, birth weight for gestational age and postnatal age at blood sample) and for maternal characteristics (age, parity, BMI, smoking, diabetes, and hypertensive disease).
